# Ovario- protective effect of *Moringa oleifera* leaf extract against cyclophosphamide-induced oxidative ovarian damage and reproductive dysfunction in female rats

**DOI:** 10.1038/s41598-024-82921-7

**Published:** 2025-01-07

**Authors:** Seham Samir Soliman, Ahmed A. Suliman, Khaled Fathy, Ahmed A. Sedik

**Affiliations:** 1 Department of Animal Reproduction and Artificial Insemination, Veterinary Research Institute, National Research Centre, Giza, 12622 Egypt; 2 Horticultural Crop Technology Department, Agriculture Research Institute, National Research Centre, Giza, 12622 Egypt; 3 Electron Microscopy Unit, Mansoura University, El Mansoura, 35516 Egypt; 4 Pharmacology Department, Medical Research and Clinical Studies Institute, National Research Centre, Giza, 12622 Egypt

**Keywords:** Cyclophosphamide, *Moringa oleifera*, Ovarian function, Oxidative stress, Pro-inflammatory mediators, Biochemistry, Diseases

## Abstract

It is crucial to develop new tactics to prevent ovarian tissue damage in women whose reproductive toxicity is caused by chemotherapy. The present investigation was performed to assess the protective effects of *Moringa oleifera (M. oleifera)* leaf extract on cyclophosphamide (CP)-induced ovarian damage and reproductive dysfunction. Thirty-two female, healthy Wistar albino rats were randomly assigned to four groups (8 rats/group). The first group was given saline intraperitoneally (i.p.). The second group was given a single dose of CP (200 mg/kg; i.p.). The third and fourth groups were given *M. oleifera* leaf extract (150 and 250 mg/kg; orally) for 20 days before receiving CP on the final day of the experiment. Hormonal assessments, including follicle stimulating hormone (FSH), luteinizing hormone (LH), and estrogen (ES), were performed 24 h after CP administration. In addition, the antioxidant status and inflammatory response against CP were evaluated. Moreover, detailed histopathological and ultra- structural observations were conducted. For evaluation of statistical significance between different groups; One-way analysis of variance (ANOVA) with Tukey’s post hoc test was adopted. Our findings revealed that rats subjected to CP showed increased levels of FSH, LH, malondialdehyde (MDA), tumor necrosis factor-alpha, and interleukin-8 and decreased levels of ES and glutathione. Pre-treatment with *M. oleifera* leaf extract (250 mg/kg; orally) was statistically significant (p values < 0.05) as it could improve hormonal changes, oxidative stress indices, and pro- inflammatory mediator levels. Consequently, a marked improvement was observed in the ovarian and uterine architectures, with a normal ovarian reserve and a normal endothelium with normal tubular glands. In conclusion, *M. oleifera* leaf extract (250 mg/kg) could be used as a pharmaceutical supplement because it protects female rats against CP-induced ovarian damage and reproductive dysfunction.

## Introduction

Cancer is a significant global health issue and is responsible for 7.6 million deaths annually, accounting for 13% of all deaths. The estimated death rate of cancer patients may further exceed 13.1 million by the year 2030^1^. Cyclophosphamide (CP) is widely utilized as a prominent alkylating agent in the treatment of cancer because of its remarkable impact on the immune system^2^. CP is commonly prescribed for patients diagnosed with lymphoma, multiple myeloma, leukemia, ovarian cancer, breast cancer, small cell lung cancer, neuroblastoma, or sarcoma^3^. Nevertheless, the therapeutic application of CP is limited as a result of its detrimental consequences, such as alopecia, nausea, vomiting, nephrotoxicity, hepatotoxicity, neurotoxicity, cardiotoxicity, immunogenicity and bone marrow suppression^4^. In addition, previous studies have documented the adverse reproductive effects of CP in female patients such as ovarian dysfunction, genetic abnormalities, chromosomal disruptions, aneuploid in somatic cells, and destruction of primary follicles, which can cause infertility and pre- mature menopause among women^5–7^. As a result, it is critical to discover new effective compounds derived from natural sources to reduce the side reproductive effects of CP^8,9^

Medicinal plants include many phytochemical antioxidants, which are popular because of their high safety margin, exceptional efficacy, and low cost^10,11^. Among them,* Moringa oleifera (M. oleifera)* is the predominant- cultivated plant from the monogeneric family, the Moringaceae, which is globally distributed across many tropical and subtropical regions^12^. The leaves of *M. oleifera* have been shown to be a significant reservoir of essential macronutrients and micronutrients. They are particularly abundant in several minerals, such as thiamine, riboflavin, niacin, protein, and vitamin A, B-complex, and C^13^. *M. oleifera* exhibits diverse pharmacological activities such as anticancer and ulcer-healing effects^14–16^. According to previous studies, *M. oleifera* has the potential to increase follicle formation in rats with polycystic ovarian syndrome (PCOS) and promote oocyte maturation in sheep^17,18^. To the best of our knowledge, our study is the first to elucidate the protective effect of *M. oleifera* leaf extract against CP-induced reproductive dysfunction in female rats on the basis of several hormonal, biochemical, histopathological and ultrastructural observations.

## Materials and methods

### *M. oleifera* leaf extraction

*M. oleifera* dry leaves were obtained from the *Moringa* planting unit of the Scientific Association of Moringa (National Research Centre, Dokki, Egypt). The leaves of *M. oleifera* were immersed in water for 15 min and dehydrated via an air dryer (55 °C). The samples were subsequently crushed into a fine powder via a household grinder. The resulting powder was then sifted through 60-mesh sieves to ensure a uniform particle size. Finally, the powdered leaves were kept at 7 °C. The preparation of the aqueous extract from *M. oleifera* leaves involved homogenizing 40 g of dry powder with 100 ml of hot water. The mixture was then allowed to sit at room temperature for 24 h, with continuous stirring with a glass rod. The filtrate was filtered through a Whatman No. 1 filter. The filtrate was concentrated by using a rotary evaporator, resulting in a reduction to 8% of its original volume at a temperature of 55 °C^19^.

### Chemicals

Cyclophosphamide was purchased from Sigma-Aldrich, USA (CAS number: 605–19-2; 99.7% pure). A previous study reported that the oral LD_50_ of *M. oleifera* leaf extract was 3000 mg/kg^20^.

### Experimental animals

Mature female Wistar albino rats, weighing between 200–225 g and ranging in age from 6 to 8 weeks, were supplied by the animal breeding colony of the National Research Centre (Egypt). The rats were provided with a typical rat diet and were given unrestricted access to water. The study received approval from the Medical Research Ethics Committee (MREC) of the National Research Centre, with the assigned approval number being 2411022023 and all methods were performed in accordance with the relevant scientific guidelines and regulations. The current study is reported in accordance with ARRIVE guidelines.

### Experimental groups

Thirty-two female cycled rats were subjected to a one-week acclimatization period. The rats were randomly separated into four groups (8 rats/ group).

- The first group was given physiological saline; 1 ml/kg, i.p. thought the experiment.
- The second group was given cyclophosphamide (200 mg/kg, i.p. single dose)^21^.
- The third group was orally administered *M. oleifera* leaf extract (150 mg/kg) for 20 days, after being dissolved in saline followed by i.p. injection of CP on the last day of the experiment^22^.
- The fourth group was orally administered *M. oleifera* leaf extract (250 mg/kg; orally), after being dissolved in saline for 20 days followed by i.p. CP on the last day of the experiment^23^.

At the end of the study, the weights of both the rats and their respective reproductive tracts were measured. To verify synchronization of the estrous cycle, a vaginal swab was used. The samples were fixed in a 96% ethanol solution, and then stained with hematoxylin and eosin^24^. After anaesthesia with ketamine (100 mg/kg) and xylazine (10 mg/kg), blood samples were collected from the eyes of the retro-orbital plexus of the rats^25^. Sera were separated by centrifugation at 3000 rpm for 15 minutes at 4 °C (Laborezentrifuger, cooling centrifuge, 2k15, Sigma, Germany) to monitor follicle stimulating hormone (FSH), luteinizing hormone (LH), and estrogen (ES) levels. The rats were euthanized through cervical dislocation, followed by a ventral midline incision to reveal their reproductive organs. The genital tract and ovaries were then dissected, weighed and homogenized to evaluate the oxidative stress indices and the levels of proinflammatory cytokines. In addition, histopathology and electron microscopy studies were conducted by keeping the tissue in 10% formalin buffer and 4% glutaraldehyde, respectively^26^.

#### Phytochemical analysis

##### Total phenolic content (TPC)

The evaluation of the TPC was conducted via the Folin-Ciocalteu technique. In a concise manner, 50 μL of the extract was transferred via a pipette into a test tube and thereafter diluted to a final volume of 3.5 mL by adding distilled water. A volume of 250 μL of Folin- Ciocalteu reagent was subsequently introduced. Following a period of 5 min, the combination underwent neutralization with the addition of 1.25 mL of a sodium carbonate (Na2CO3) solution with a concentration of 20%. The absorbance was measured at a wavelength of 725 nm, using the solvent blank as a reference, following a 40-min incubation period in darkness at room temperature. The quantification of the overall phenolic content was conducted via a calibration curve that was produced via the addition of chlorogenic acid. The results are presented as milligrams of chlorogenic acid equivalent (mg/ CAE) per gram of the *moringa* bean sample^27^.

#### Antioxidant assays

##### Determination of radical *2*,*2*- diphenyl-1-picrylhydrazyl* (*DPPH) and 2,2′-azinobis-[3- ethylbenzothiazoline- 6-sulfonic acid (ABTS) scavenging capacity

Stable DPPH was used to assess the free radical scavenging activity of *M. oleifera* leaf extracts. Fifty microlitres of the extracts were aliquoted and subsequently combined with 2.95 millilitres of a 200 micromolar solution of DPPH. The absorbance was measured at a wavelength of 517 nm, using pure methanol as the reference, following a 1 h incubation period in the absence of light. The standard curve was developed with Trolox as the reference compound. The outcomes were quantified in terms of milligrams of Trolox equivalent (mg TE) per gram of *moringa* sample.

The stock solutions of ABTS were prepared by combining equal volumes of a 7 mM aqueous solution of ABTS with a 2.45 mM solution of potassium persulfate and allowing the reaction to proceed for 16 h at room temperature in the absence of light. The solution was made by diluting 1 mL of ABTS solution with 60 mL of ethanol: water (50:50, v/v) to achieve an absorbance of 1.0 ± 0.02 units at a wavelength of 734 nm via a spectrophotometer. Fifty microliters of the extracts were combined with 4.95 millilitres of the ABTS solution and allowed to react for one hour in the dark. The absorbance was subsequently measured at a wavelength of 734 nm. The standard curve was developed with Trolox as the reference compound. The data are expressed as milligrams of Trolox equivalent (mg TE) per gram of moringa bean sample^28^.

#### High-performance liquid chromatography (HPLC) analysis of *M. oleifera* leaf extracts

Chromatographic analysis of M. oleifera leaf extracts were performed with an HPLC model 1100 (Agilent Technologies, CA, USA) system outfitted with a quaternary pump, an autosampler injector, and a diode array detector (DAD). The target compounds in the extract were identified and quantified by matching their retention times and peak areas to those of the standards, as described by Kim et al. (2006) with some modifications^29^.

#### Measurement of live weight changes

The live weights of each rat were measured via a weighing scale and are expressed as *the means* ± *SEs*. This procedure was conducted at the onset of the study and on a weekly basis until the end of the study. Furthermore, the weight of their reproductive tract was also measured.

#### Biochemical indices

##### Evaluation of the serum follicle-stimulating hormone (FSH), luteinizing hormone (LH), and estradiol (ES) levels

The levels of follicle-stimulating hormone (FSH), luteinizing hormone (LH), and estradiol (ES) in the serum were assessed via enzyme-linked immunosorbent assay (ELISA) kits provided by Chemux Bioscience, Inc., USA, following the instructions provided by the manufacturer (CAT: DL-FSH-Ra, DL-LH-Ra, and K030-H, respectively).

##### Evaluation of the ovarian values of reduced glutathione (GSH)

The ovarian levels of GSH were assessed via Bio- diagnostic kits from Egypt (CAT: GR 25 11) by using spectrophotometer (Model. V630, Japan)^30,31^.

##### Evaluation of the ovarian value of malondialdehyde (MDA)

The levels of MDA in the ovaries were assessed via Bio- diagnostic kits from Egypt (CAT: MD 25 29)^32,33^.

##### Evaluation of the ovarian levels of proinflammatory cytokines

The levels of TNF-α and IL-8 in the ovaries were quantified via enzyme-linked immunosorbent assay (ELISA) kits (USCN, Wuhan, China). The measurements were conducted according to the manufacturer’s instructions at an optical density of 450 nm. The obtained data are reported in pg/mL CAT: SEA133Rb and SEA080Hu, respectively)^34^.

#### Histopathological examination and ovarian and uterine morphometry

The isolated ovaries and uterus were preserved in 70% ethyl alcohol after being fixed in 10% formalin. The specimens were cleared in xylene, embedded in paraffin, sectioned and stained with hematoxylin and eosin^35^. Total ovarian score damage was recorded using a graduated scale (0 = none; 1 = mild; 2 = moderate; 3 = sever) by a blind pathologist.

#### Ovarian follicle counts

The ovarian sections were photographed via an MC Series Digital Microscope camera—Am scope, which was connected to a compound light microscope (Olympus microscope CX31) with a 10X objective. A methodical approach was employed to capture three to five photos of each ovarian segment, which were arranged in rows from left to right and top to bottom. The captured images encompassed the whole area of each ovarian segment, with very little tissue exclusion. The images of each segment were accessed via Image-Pro software (Media Cybernetics, Bethesda, MD, USA).

#### Ultrastructural examination of the ovary via transmission electron microscopy

Ovarian samples from the experimental groups were fixed in 4F1G (4% formaldehyde and 1% glutaraldehyde) fixative for at least 2 h overnight, according to Cheville 2014. The samples were immersed in 8% (0.2 M) sucrose in 0.1 M PB 3 × 15 min or overnight and then fixed in 1% osmium tetroxide in 0.1 M PB for 1 h. The samples were subsequently immersed in 8% (0.2 M) sucrose in 0.1 M PB for 3 × 15 min. The samples were dehydrated via immersion in 50% ethanol for 15 min, 70% ethanol for 15 min, 95% ethanol for 15 min, 100% ethanol for 2 × 15 min, and 100% acetone for 1 h. The samples were subsequently embedded by immersion in 1:1 EMBed 812 and propylene oxide for 1–2 h, followed by 2:1 EMBed 812: propylene oxide overnight in desiccators with the top off ready in the form of beam capsules.

The last step involved sectioning the samples by cutting them into thick Sects. (0.5–1.0 µm) on a slide and allowing them to dry on a slide warmer. Toluidine blue stain was applied for 2–5 min. Then, the sections were examined under a microscope to determine the exact spot for cutting ultrathin sections that were 60–90 nm thick. Finally, all the sections were gathered into grids. The ultrathin sections were analyzed via transmission electron microscopy (JEOL 2100) at Mansoura University, operating at a voltage of 160 kV^36^.

## Statistical analysis

The results are reported as the means ± SDs (8 rats). We used one-way analysis of variance (ANOVA) with Tukey’s post hoc test between the groups. Prior to performing ANOVA, all samples were subjected to normality testing via the Shapiro‒Wilk test. The results were statistically significant (p values < 0.05). GraphPad Prism v. 8.0 software developed by GraphPad Software, Inc., USA, was used to analyze the data.

## Results

### Total phenolic content and antioxidant capacity of *M. oleifera* leaf extract

The data are presented in the Table 1 shows the effects of different concentrations of *M. oleifera* leaf extract on the antioxidant activity as well as the total phenolic content of the leaf extract. The results clearly revealed that the highest values of antioxidant activity and total phenolic count were recorded when compared with those of the control.

**Table 1:** Representing total phenolic contents and antioxidant capacity of *Moringa oleifera* extract.

Sample	Total phenols (mg GAE/ml)	Total flavonoids (mg CE/ml)	DPPH (mg TE/ml)	ABTS (mg TE/ml)
*Moringa* leaf extract	0.61	0.59	0.85	0.66

*GAE*  gallic acid equiv., *CE* catechin equiv., *TE* trolox equiv.

### High-performance liquid chromatography (HPLC) values of *M. oleifera* leaf extract

The HPLC profiles of the extracts were analyzed for 22 gallic acid, protocatechuic acid, p-hydroxybenzoic acid, catechin, chlorogenic acid, caffeic acid, syringic acid, vanillic acid, ferulic acid, sinapic acid, p-coumaric acid, rutin, hisperdin, rosmarinic acid, apegnin-7-glycoside, oleuropein, diodzein, genistin, cinnamic acid, quercetin, kaempferol and chrysin. The quantitative results are shown in the (Table 2). For the extract, seven major phenolics were observed at concentrations of 2.238, 7.875, 28.432, 2.929, 94.664, 7.28 and 55.960 μg/g for gallic acid, p-hydroxybenzoic acid, chlorogenic acid, vanillic acid, rutin, rosmarinic acid and apegnin-7-glycoside, respectively. Although rutin and apegnin-7-glycoside had the highest concentrations in the extract (94.664 and 55.960 g/g, respectively), the phenolic compound with the lowest concentration was gallic acid (2.238 μg/g).

**Table 2:** Representing total phenolic contents and antioxidant capacity of *Moringa oleifera* leaf extract.

	Compound	Tr (min)	Conc (ugml)
1	Gallic acid	4.1	2.238
2	Protocatechuic acid	6.8	ND
3	*p*-hydroxybenzoic acid	10.3	7.875
4	Catechin	12	ND
5	Chlorogenic acid	13.2	28.432
6	Caffeic acid	13.6	ND
7	Syringic acid	15.1	ND
8	Vanillic acid	16.3	2.929
9	Ferulic acid	20.77	ND
10	Sinapic acid	21.5	ND
11	*p*-coumaric acid	26.1	ND
12	Rutin	24.9	94.664
13	hisperdin		ND
14	rosmarinic acid	30.3	7.28
15	apegnin-7-glycoside	29.2	55.960
16	Oleuropein	32.9	ND
17	diodzein	34	ND
18	genistin	39	ND
19	Cinnamic acid	35.5	ND
20	quercetin	36.1	ND
21	Kaempferol	40.5	ND
22	Chrysin	53	ND

### Changes in animal weight in the studied groups

According to the Table 3, the CP group had the lowest final body weight and calculated weight gain and the control group had the highest. CP-treated female rats revealed a decrease in the final body weight by 74%, as compared with control group. While CP rats received *M. oleifera* 150&250 mg/kg) showed significantly an increase in in the final body weight by 1.2fold and 1.34fold, respectively, as compared with CP group.

**Table 3:** Effect of administration of *Moringa oleifera* on the values of initial & final body weight and reproductive tract weight in female rats received CP induced- reproductive dysfunction.

Groups	Initial body weight (g)	Final body weight (g)	Weight gain (g)	Reproductive tract weight (g)
Control	133.3 ± 2.6	175.5 ± 2.1	42.2 ± 2.5	0.45 ± 0.01
CP(200 mg/kg) group	137.5 ± 5.1*	130.7 ± 2.3*	−7.2 ± 2.8*	0.23 ± 0.01*
CP + *Moringa oleifera* (150 mg/kg)	135.5 ± 4.4*^@^	157 ± 2.3*^@^	21.5 ± 2.1*^@^	0.54 ± 0.05*^@^
CP + *Moringa oleifera* (250 mg/kg)	136.5 ± 3.5*^@^	176 ± 7.2*^@^	39.5 ± 3.7*^@^	0.82 ± 0.1*^@^

Reproductive dysfunction was induced in female rats by i.p. administration of CP (200 mg/kg. b.w.). Rats were treated with *M. oleifera* leaf extract (150 and 250 mg/kg; orally). 24 h after the last dose of CP, the weights of both the rats and their respective reproductive tracts were measured. Results are expressed as mean ± SD (n = 8). *Significant difference from control group, p < 0.05. @ Significant difference from CP- received group, *P* < *0.05.*

### Photographs of the genital tract from different groups

**Fig. 1:**
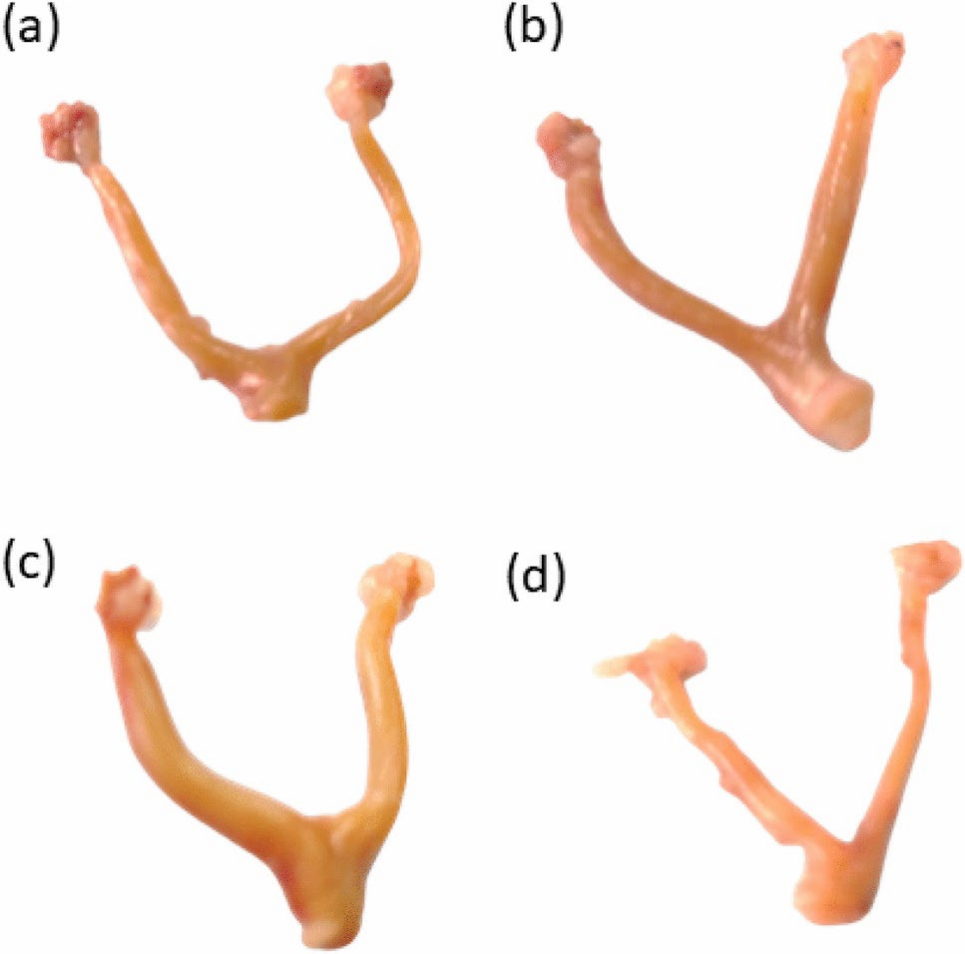
Effect of administration of *M. oleifera* leaf extract on the photographs of the genital tract of female rats received CP induced reproductive dysfunction. Reproductive dysfunction was induced in female rats by i.p. administration of CP (200 mg/kg. b.w.). Rats were treated with *M. oleifera* leaf extract (150 and 250 mg/kg; orally). At the end of the study, rats were sacrificed by cervical dislocation and their genital tract was weighted and photographed. Where (a; control, b; CP group, c; CP + *M. oleifera* (150 mg/kg) group, d; CP + *M. oleifera* (250 mg/kg) group.

According to Fig. 1, the photographs of the genital tract (ovarian and uterine tissues) were significantly improved in all groups except the CP group. Rats that received (CP + *M. oleifera* 150 mg/kg) showed improvements in the general characteristics of the genital tract (ovarian and uterine). The ovarian and uterine tissues of the rats that received (CP + *M. oleifera* 250 mg/kg) revealed a normal architecture resembling that of the control animals.

### Effect of *M. oleifera* leaf extract on FSH and LH values in female rats with CP -induced reproductive dysfunction

**Fig. 2:**
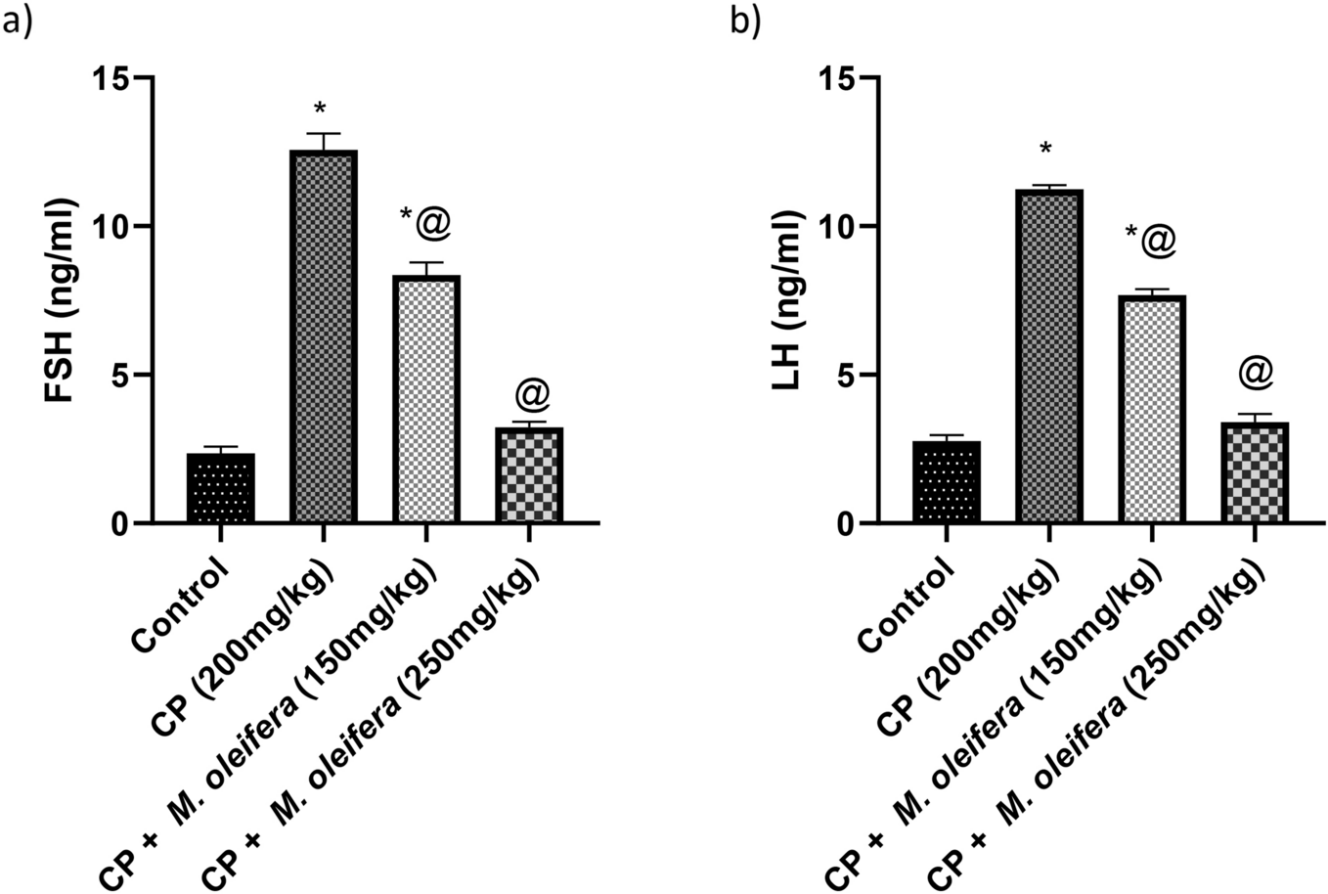
Effect of administration of *M. oleifera* leaf extract on the FSH and LH values in female rats received CP induced reproductive dysfunction. Reproductive dysfunction was induced in female rats by i.p. administration of CP (200 mg/kg. b.w.). Rats were treated with *M. oleifera* leaf extract (150 and 250 mg/kg; orally). 24 h after the last dose of CP, serum levels of FSH and LH were evaluated. Results are expressed as mean ± SD (n = 8). *Significant difference from control group, p < 0.05. @ Significant difference from CP- received group, *P* < *0.05*.

Compared with control rats, CP-treated female rats presented an increase in the concentration of FSH, which was approximately fivefold greater. However, the CP rats that were orally administered two doses of *M. oleifera* leaf extract (150 and 250 mg/kg) presented a significant reduction in the concentration of FSH, which was approximately 0.6-fold and 0.25-fold greater than that of the CP group (Fig. 2a).

The concentration of LH in female rats that received CP was significantly increased by fourfold. However, the CP rats that were orally administered two doses of *M. oleifera* leaf extract (150 and 250 mg/kg) presented a significant reduction in the concentration of LH, which was approximately 0.7- and 0.30-fold greater than that in the CP group, respectively (Fig. 2b).

### Effect of *M. oleifera* leaf extract on the concentration of ES in female rats with CP-induced reproductive dysfunction

**Fig. 3:**
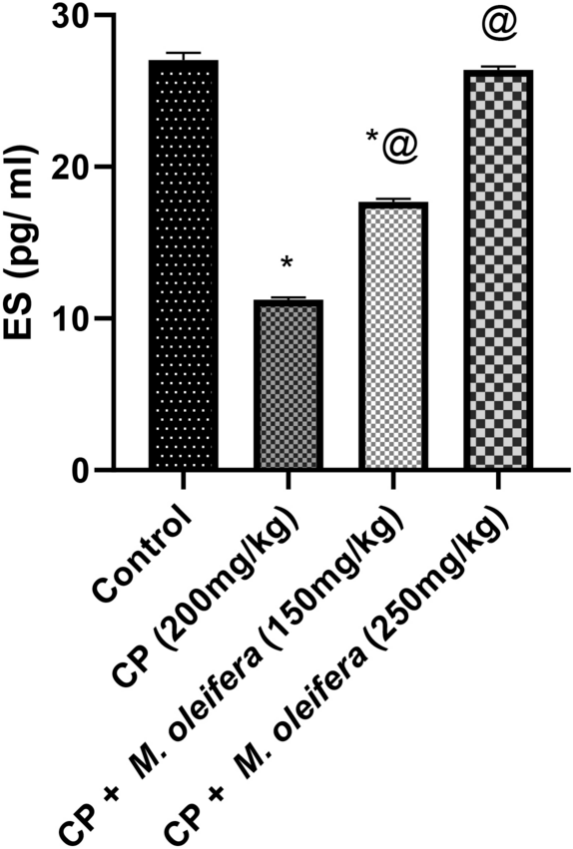
Effect of administration of *M. oleifera* leaf extract on the ES values in female rats received CP induced reproductive dysfunction. Reproductive dysfunction was induced in female rats by i.p. administration of CP (200 mg/kg. b.w.). Rats were treated with *M. oleifera* leaf extract (150 and 250 mg/kg; orally). 24 h after the last dose of CP, serum levels of ES were evaluated. Results are expressed as mean ± SD (n = 8). *Significant difference from control group, p < 0.05. @ Significant difference from CP- received group, *P* < *0.05*.

The concentration of ES in female rats that received CP was significantly lower (0.4-fold) than that in control rats. However, the CP rats that were orally administered two doses of *M. oleifera* leaf extract (150 and 250 mg/kg) presented significant increases in the concentration of ES, which reached approximately 1.6-fold and 2.4-fold greater than those in the CP group, respectively (Fig. 3).

### Effect of *M. oleifera* leaf extract on the GSH and MDA values of female rats with CP -induced reproductive dysfunction

**Fig. 4:**
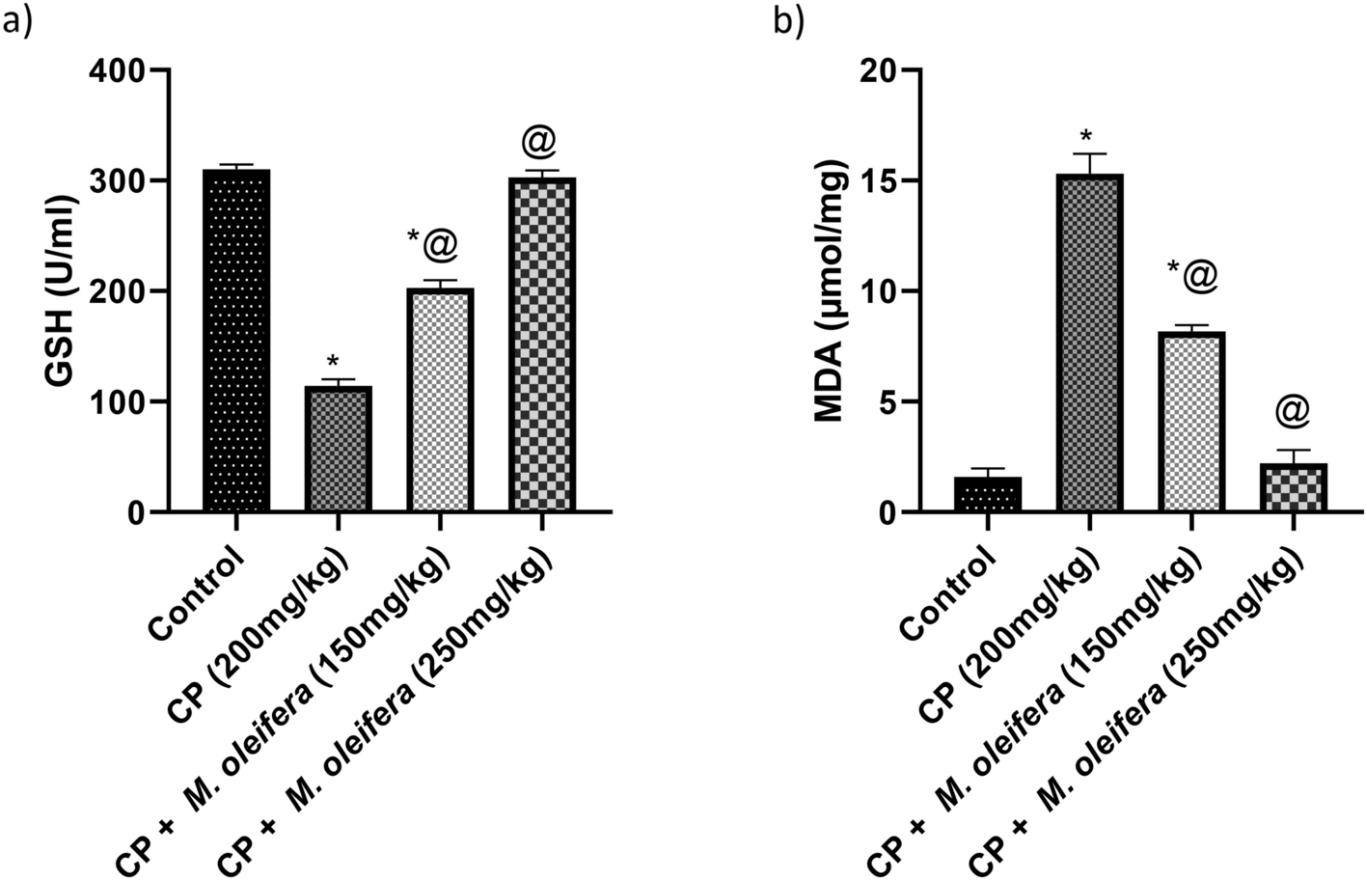
Effect of administration of *M. oleifera* leaf extract on GSH and MDA values in female rats received CP induced reproductive dysfunction. Reproductive dysfunction was induced in female rats by i.p. administration of CP (200 mg/kg. b.w.). Rats were treated with *M. oleifera* leaf extract (150 and 250 mg/kg; orally). 24 h after the last dose of CP, ovarian levels of GSH and MDA were evaluated. Results are expressed as mean ± SD (n = 8). *Significant difference from control group, p < 0.05. @ Significant difference from CP- received group, *P* < *0.05*.

Compared with those in normal rats, the levels of GSH and MDA in female rats that received CP were significantly decreased by 0.3-fold and increased by ninefold, respectively. The rats in the CP + *M. oleifera*; 150 mg/kg) significantly increased the concentration of GSH, reaching approximately 1.57-fold greater than that in the CP group. Moreover, they were related to a significant reduction in the values of MDA, reaching approximately 0.53-fold. The rats in the CP + *M. oleifera*; 250 mg/kg) restored the levels of GSH and MDA (Fig. 4a,b).

### Effect of *M. oleifera *leaf extract on TNF-*α* and IL-8 levels in female rats with CPinduced reproductive dysfunction

**Fig. 5:**
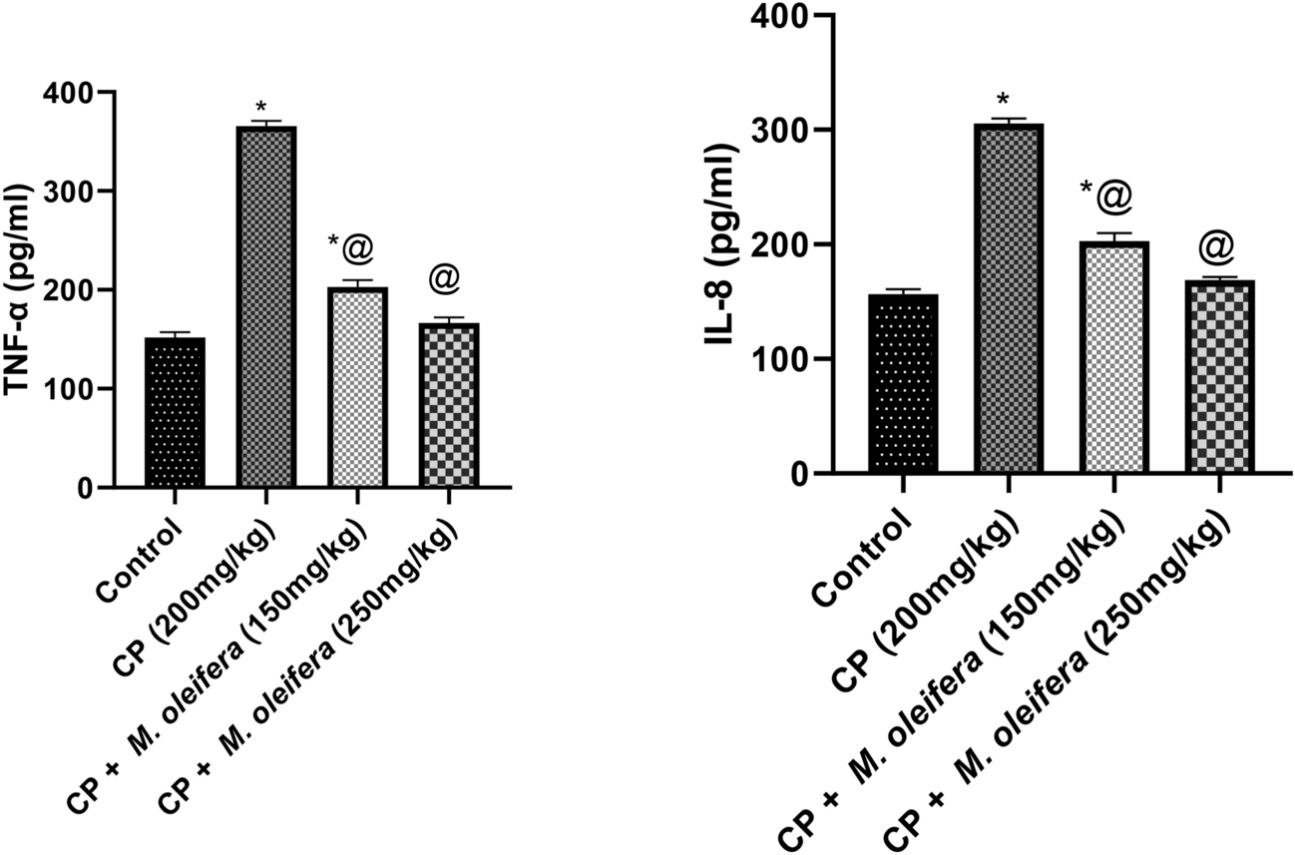
Effect of administration of *M. oleifera* leaf extract on TNF-*α* and IL-8 values in female rats received CYP induced reproductive dysfunction. Reproductive dysfunction was induced in female rats by i.p. administration of CP (200 mg/kg. b.w.). Rats were treated with *M. oleifera* leaf extract (150 and 250 mg/kg; orally). 24 h after the last dose of CP, ovarian levels of TNF-*α* and IL-8 were evaluated. Results are expressed as mean ± SD (n = 8). *Significant difference from control group, p < 0.05. @ Significant difference from CP- received group, *P* < *0.05*.

Compared with the control rats, the CP-treated rats presented increases in the levels of TNF-*α* and IL-8 of 250 and 190%, respectively. Rats received CP + *M. oleifera*; 150 mg/kg) significantly showed a decrease in the levels of TNF-*α* and IL-8 to approximately 0.5-fold and 0.6-fold, respectively, as compared with CP group. While, rats in the CP + *M. oleifera*; 250 mg/kg) significantly revealed a marked decrease in the levels of TNF-*α* and IL-8 to approximately 0.4-fold and 0.5-fold, respectively, as compared with CP group (Fig. 5a,b).

### Histopathology of the ovaries and uterus

#### Ovarian sections

**Fig. 6:**
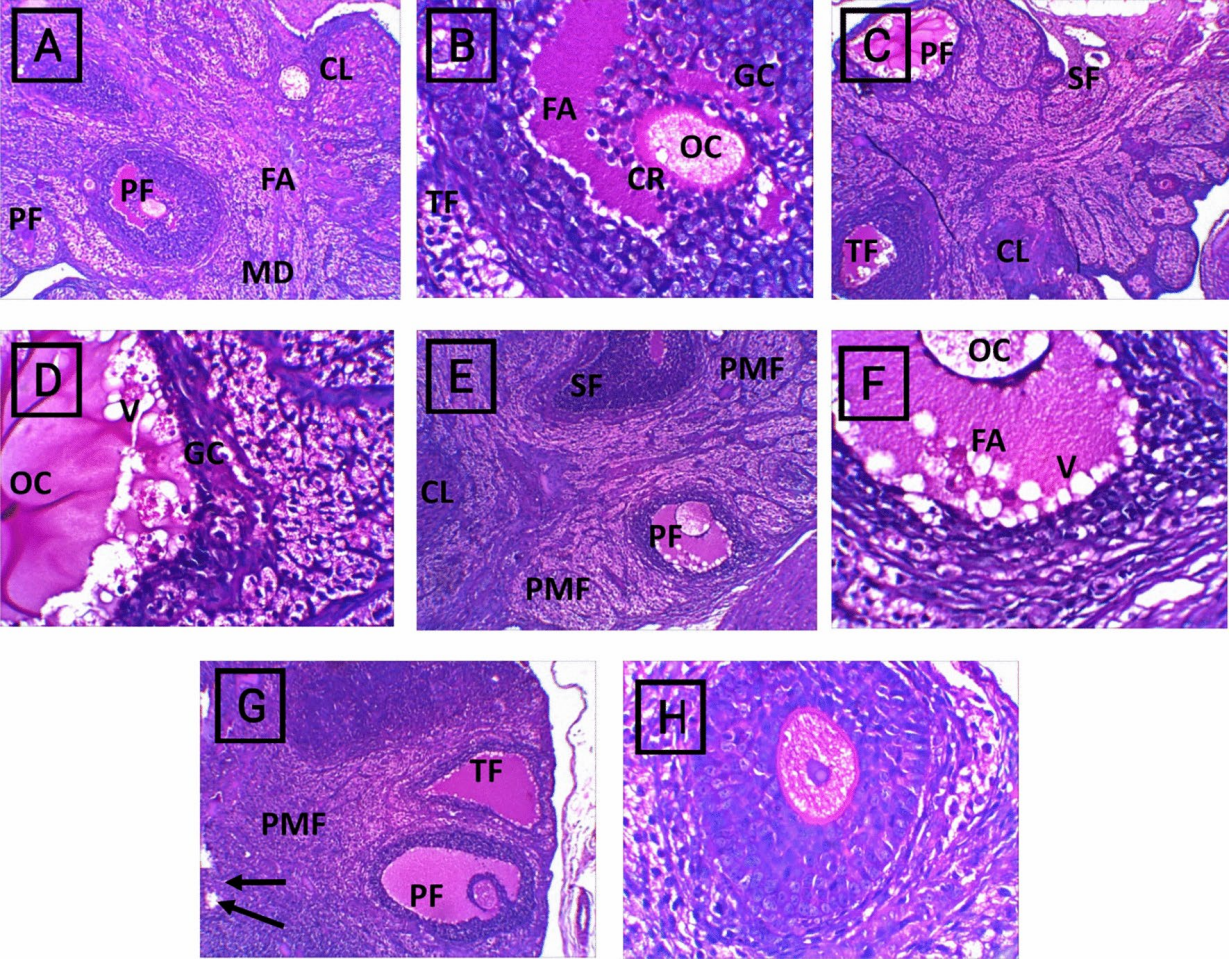
Photomicrographs of H&E staining of ovarian sections of control and different experimental groups, (**A**,**B**) Control group. (**A**) showed normal ovarian histoarchitecture, normal growing follicles till normal preovulatory follicles (PF), normal Medulla (MD) and corpus luteum (CL) can also be seen.(**B**) Showed normal preovulatory follicle are seen with large follicular antrum (FA) and corona radiate (CR) surrounding the oocyte (OC). A normal cortical stroma can be seen with the theca folliculi (TF) surrounding the granulosa cells (GC). (**C**,**D**) Cyclophosphamide group. (**C**) exhibited apoptotic signs are seen on the antral follicles as in degenerating (atretic) tertiary follicle (AF) of with distorted granulosa cells (GC) and in damage preovulatory follicle (PF), completely atretic secondary follicle (SF), with the appearance fibrotic corpus luteum (CL). (D) Preovulatory follicle show vacuolated oocyte surrounded by fibrotic granulosa cells (GC). (**E**,**F**) CP + *M. oleifera* 150 mg/kg group showed mild damage of the ovarian tissue, primordial follicle (PMF) appear normal, the secondary follicle (SF) with normal size and abnormal shape. (**F**) Preovulatory follicle (PF) still with few signs of apoptosis as unhealthy oocyte (OC) and vacuolated (V) follicular antrum (FA). (**G**,**H**) CP + *M. oleifera* 250 mg/kg (**G**) showed most ovarian tissue preserves its normal histological appearance with normal growing cells as: primordial follicle (PMF), secondary follicle (SF) and Preovulatory follicle (PF), with presence of few vacuolated follicles (arrow). (**H**) Nearly normal Preovulatory follicle with typical structure. (Magnification: (**A**,**C**,**E**,**G**) 10X and (B,D,F,H) 40X).

The control group displayed a normal ovarian appearance with normal growing follicles. The cyclophosphamide group exhibited apoptotic signs on the antral follicles and distorted granulosa cells with the appearance of a fibrotic corpus luteum. The rats in the CP + *M. oleifera* 150 mg/kg group presented mild damage to the ovarian tissue, as evidenced by the appearance of few signs of apoptosis in preovulatory follicles. In contrast, in the rats that received 250 mg/kg CP + *M. oleifera*, most of the ovarian tissue preserved its normal histological appearance with normal growing cells (Fig. 6).

#### Uterus sections

**Fig. 7:**
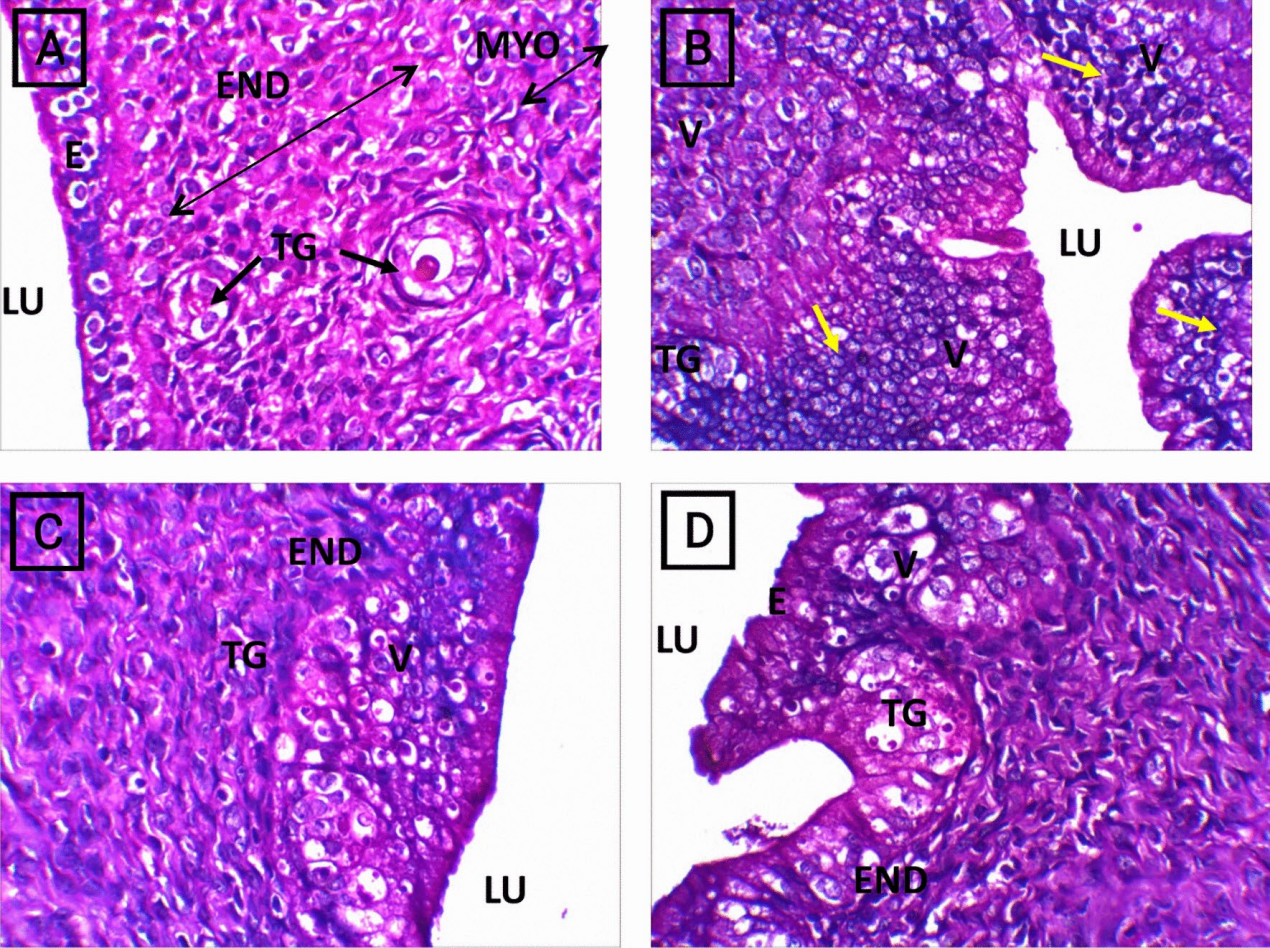
Photomicrographs of H&E staining of uterus sections of control and different experimental groups, (**A**) group Control group showed normal uterine architecture presented with intact endothelium (E) , normal tubular glands (TG) and patent wide uterine Lumen (UL) with different layers; endometrium (End), myometrium (myo) with normal thickness (double head arrows). (**B**) CP (200 mg/kg) group exhibited noticed uterine section degeneration, appear of massive leukocyte infiltration (Yellow Arrow), congested tubular glands (TG), multi vacuoles (V), and endothelium with apoptotic epithelium cells. (**C**) CP + *M. oleifera* 150 mg/kg group showi mildly disrupted uterine appearance, endometrium (End) without infiltration, few epithelial vacuolar degeneration (V) and slight Congested tubular gland (TG). (**D**) CP + *M. oleifera* 250 mg/kg showed marked Improvement of uterine section architecture, normal endothelium (**E**), normal tubular glands (TG) and few vacuoles (V) in endometrium (End). (Magnification 40X).

The control group presented a normal uterine architecture with an intact endothelium, normal tubular glands and a patent wide uterine lumen with normal thickness. The CP (200 mg/kg) group exhibited uterine section degeneration, massive leukocyte infiltration, congested tubular glands, multiple vacuoles, and an endothelium with apoptotic epithelial cells. The rats in the CP + *M. oleifera* 150 mg/kg group showed a mildly disrupted uterine appearance, few cases of epithelial vacuolar degeneration and slightly congested tubular glands. The rats that received 250 mg/kg CP + *M. oleifera* exhibited marked improvements in uterine section architecture, normal endothelium and normal tubular glands (Fig. 7).

### Effects of *M. oleifera* administration on changes in the ovarian follicles of female rats with CPinduced reproductive dysfunction

The mean number of follicles in control group was primordial (2.25 ± 0.05), primary (4.5 ± 0.08), and secondary (6.75 ± 0.35) and Graafian (5.75 ± 0.45) follicles. While, the ovaries of the CP-treated rats revealed degenerated tissues and reduced numbers of follicles in their ovarian tissue, where the mean numbers of follicles were primordial (1 ± 0.03), primary (1.25 ± 0.05), and secondary (1.75 ± 0.09) and Graafian (2 ± 0.06) follicles.

Rats that received CP + *M. oleifera (*150 mg/kg) displayed normal ovarian architecture with multiple follicles, and the mean numbers of follicles were primordial (1.25 ± 0.31), primary (3 ± 0.02), and secondary follicles (4 ± 0.63) and Graafian follicles (3.5 ± 0.05). The mean ovarian architecture of the rats given CP + *M. oleifera (*250 mg/kg) was primordial (2 ± 0.4), primary (4 ± 0.03), and secondary follicles (5 ± 0.015) and Graafian follicles (4.5 ± 0.92) (Table 4).

**Table 4:** Effect of administration of *M. oleifera* on the changes in ovarian follicles of female rats received CP induced- reproductive dysfunction.

Groups	Primordial follicle	Primary follicle	Secondary follicle	Graafian follicle
Control	2.25 ± 0.05	4.5 ± 0.08	6.75 ± 0.35	5.75 ± 0.45
CP (200 mg/kg) group	1 ± 0.03*	1.25 ± 0.05*	1.75 ± 0.09*	2 ± 0.06*
CP + *M. oleifera* (150 mg/kg) group	1.25 ± 0.31*^@^	3 ± 0.02*^@^	4 ± 0.63*^@^	3.5 ± 0.05*^@^
CP + *M. oleifera* (250 mg/kg) group	2 ± 0.4*^@^	4 ± 0.03*^@^	5 ± 0.015*^@^	4.5 ± 0.92*^@^

Reproductive dysfunction was induced in female rats by i.p. administration of CP (200 mg/kg. b.w.). Rats were treated with *M. oleifera* leaf extract (150 and 250 mg/kg; orally). At the end of the study, rats were sacrificed by cervical dislocation and the changes in their ovarian follicles were evaluated. Results are expressed as mean ± SD (n = 5). *Significant difference from control group, p < 0.05. @Significant difference from CP- received group, *P* < *0.01.*

### Effect of administration of *Moringa oleifera* on the histopathological scores of ovarian damage in rats received CP induced- reproductive dysfunction

The ovarian histopathological scores were significantly lower in control group. Signs of inflammation, congestion; hemorrhage and degeneration were significantly exhibited in in rats received CP. Our findings showed that there was no significant difference in the histopathological indices in CP rat received either* M. oleifera (*150 mg/kg) or *M. oleifera (*250 mg/kg) (Table 5).

**Table 5:** Effect of administration of Moringa oleifera on the histopathological scores of ovarian damage in rats received CP induced- reproductive dysfunction.

Groups	Congestion	hemorrhage	Inflammation	Follicular degeneration
Control	0.00 ± 0.00	0.00 ± 0.00	0.12 ± 0.00	0.12 ± 0.00
CP(200 mg/kg) group	2.13 ± 0.34*	1.88 ± 0.61*	3.00 ± 0.00*	2.39 ± 0.73*
CP + *Moringa oleifera* (150 mg/kg)	1.11 ± 0.37*^@^	1.06 ± 0.31*^@^	1.65 ± 0.44*^@^	1.17 ± 0.41*^@^
CP + *Moringa oleifera* (250 mg/kg)	0.88 ± 0.35*^@^	0.67 ± 0.21*^@^	1.13 ± 0.82*^@^	0.75 ± 0.46*^@^

Reproductive dysfunction was induced in female rats by i.p. administration of CP (200 mg/kg. b.w.). Rats were treated with *M. oleifera* leaf extract (150 and 250 mg/kg; orally). 24 h after the last dose of CP, rats were sacrificed by cervical dislocation and the histopathological scores of ovarian damage were recorded. Results are expressed as mean ± SD (n = 5). *Significant difference from control group, p < 0.05. @ Significant difference from CP- received group, *P* < *0.01.*

### Ultrastructural picture of the ovary

**Fig. 8:**
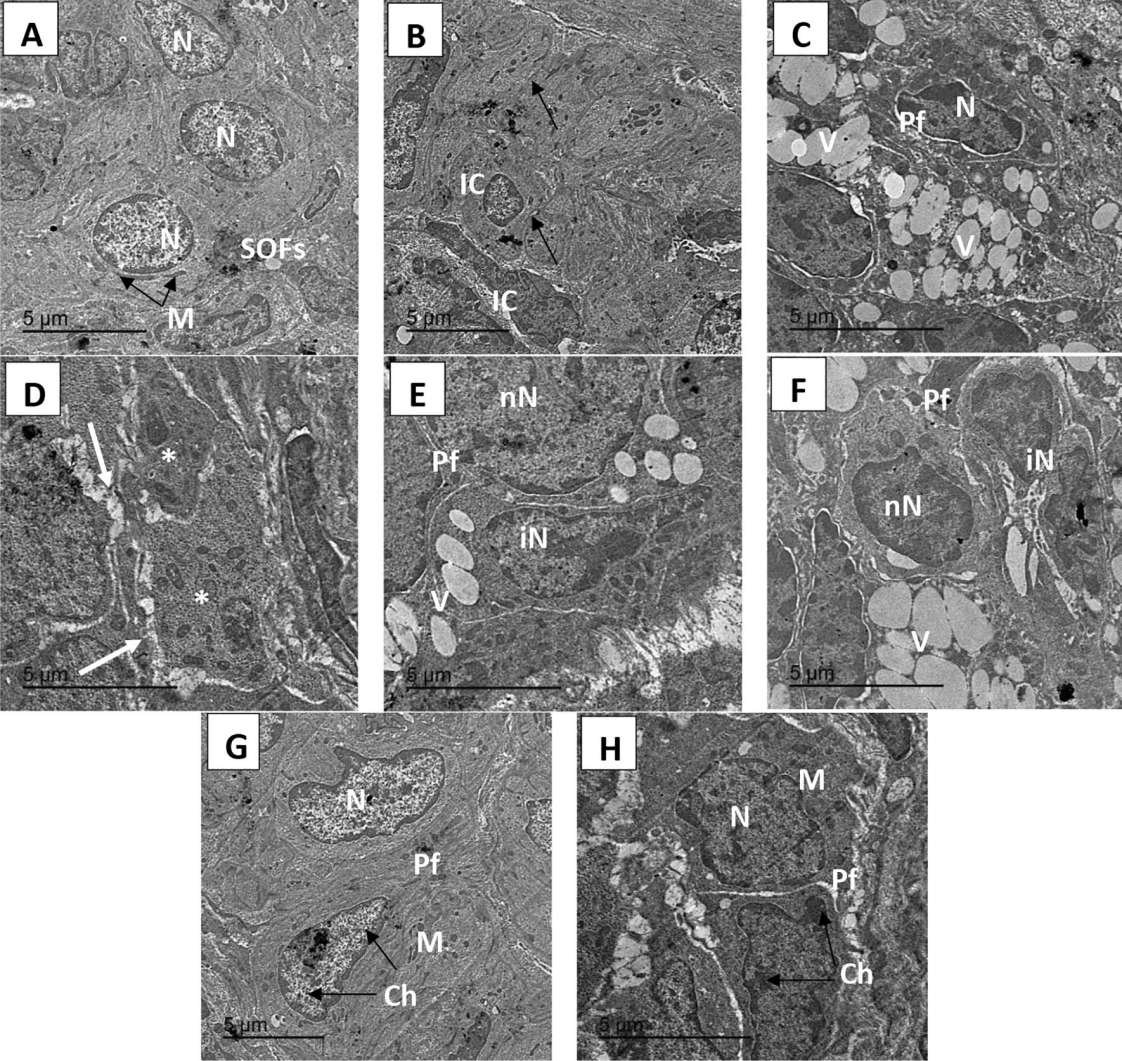
TEM micrograph of ovary from control and different treatment groups: (**A**,**B**) control group (**A**) showing a single oocyte follicle (SOFs) with a clear round shaped nucleus (N) and normal mitochondria (M) distributed throughout the cytoplasm and oriented as a grape like structure.(**B**) showing Normal ovarian interstitial cells (IC) with contacted cell–cell junction (arrow). (**C**,**D**) CP (200 mg/kg) group exhibited degeneration of primordial follicle follicles (Pf), follicles nucleus appeared shrinked losing its normal shape and structure, with appearing of multi-vaculation (V). Follicles reach to attritic phase (*), with gapping between ovarian cells (white arrow). (**E**,**F**) CP + *M. oleifera* 150 mg/kg group showing slight improvement of single oocyte follicle , where its nucleus preserve its normal round cells (NN) and other nucleus appear irregular (IN).with still appearing of little multivaculation (V) .(**G**,**H**) CP + *M. oleifera* 250 mg/kg showed ovarian tissue appear near normal to control group where the primordial follicles with normal oval shape and its nucleus appear nearly normal (N) with its normal distributed chromatin (Ch) and normal mitochondria (M).

TEM micrographs of ovaries from the different treatment groups revealed normal ovaries arranged in a grape-like structure in the normal group. In the CP group, degeneration of primordial follicles (PFs) and the appearance of multiple vacuulations were observed. The rats that received CP + *M. oleifera at* 150 mg/kg exhibited a slight improvement in the number of single oocyte follicles, with few instances of multivaculation. Compared with those in the control group, the ovarian tissue of the rats in the CP + *M. oleifera* 250 mg/kg group appeared nearly normal, whereas the primordial follicles had a normal oval shape, and their nuclei appeared nearly normal, with normally distributed chromatin and normal mitochondria (Fig. 8).

## Discussion

This study aimed to evaluate the protective effect of *M. oleifera* leaf extract on alleviating the noxious effects of CP. In our current investigation, we observed that *M. oleifera-250* inhibited the reduction in body weight of the rats. Moreover, this study revealed that *M. oleifera-*250 possesses strong antioxidant and anti-inflammatory properties by reducing the concentrations of MDA and the proinflammatory biomarkers TNF-α and IL-8 in the ovary and increasing the concentration of GSH in the ovary. Furthermore, the hormonal results, histopathology and ultrastructural view of the ovarian follicles confirmed the establishment of the estrous cycle, the reduction in atretic follicles, and the increase in primary and secondary follicles (Fig. 1).

The decrease in body weight observed in the CP group could be attributable to anorexia, implying that CP has negative effects on the gastrointestinal system or the appetite center in the hypothalamus^37,38^. On the other hand, the enhanced body weight gain observed in *M. oleifera-* 150- and M. oleifera-250-treated rats could be attributed to polyphagia produced by stimulation of the center of the hypothalamus, and a previous study confirmed our findings^39^. Thus, in this study, *M. oleifera* leaf extract (250 mg/kg) mitigated the negative effects of CP on rat feeding patterns.

Cyclophosphamide causes chromosomal damage to primordial follicles which inhibits estrogen production; in turn, increasing amounts of luteinizing hormone (LH) and follicle stimulating hormone (FSH) are released from the anterior pituitary which recruits more follicles from the ovary and prematurely depletes the follicular pool^40,41^. A previous study revealed the adverse effects of CP on folliculogenesis, which leads to irreversible damage to the ovaries^42^. Our findings showed that the rats given CP had higher levels of FSH and LH in their serum and lower levels of ES. These findings are consistent with a previous study reporting that CP consumption significantly increased LH and FSH values in female rats^43^. The number of atretic follicles significantly reduced the serum ES concentration and the number of primordial, primary, secondary, and Graafian follicles. This follicle loss is one of the primary causes of ovarian failure and infertility caused by chemotherapy, indicating that CP has a negative effect on these parameters, which is consistent with other studies^44^. Oral administration of *M. oleifera-* at 150 and 250 mg/kg was associated with a dose-dependent increase in FSH and LH and an increase in ES values, which is attributable to the high concentration of phenolic compounds and isothiocyanate in *M. oleifera* leaf extract^45,46^.

It is generally documented that oxidative stress and reactive oxygen species (ROS) have a significant effect on female reproduction and ovulation^47^. Oxidative stress arises when the generation of ROS overwhelms the ability of cells to defend themselves from increased ROS through adequate quantities of antioxidant enzymes, which are essential for ovarian function^48,49^. Oxidative stress inhibits the development of oocytes by impeding their nuclear and cytoplasmic maturation and triggering apoptosis^50^. Phosphoramide mustard, a cytotoxic metabolite of CP causes the production of ROS, resulting in lipid peroxidation and redox imbalance disruption. This leads to the apoptosis of granulosa cells and an increase in antral follicle atresia^51,52^. In our study, CP administration led to oxidative injury to lipids and proteins in the ovary, as evidenced by an increase in MDA and a decrease in GSH. In the ovary, ROS impede ovarian functions by triggering lipid peroxidation and decreasing the activity of the GSH enzyme^53^. The administration of *M. oleifera* leaf extract could reduce MDA values and potentiate the value of the antioxidative enzyme; GSH because of its presence of antioxidative compounds such as quercetin, kaempferol and chlorogenic acid^54–56^.

Inflammation is a natural biological phenomenon that occurs during ovulation. However, unregulated inflammation has detrimental impacts on the generation of hormones and the process of ovulation^57^. Acrolein, a toxic metabolite of CP causes inactivation of microsomal enzymes and results in increased generation of ROS that leads eventually to increased the expression of proinflammatory cytokines^58,59^ such as TNF-α and IL-8, which promote ovary apoptosis^60^. The current investigation revealed that rats treated with *M. oleifera* leaf extract (150 and 250 mg/kg) presented lower concentrations of TNF-α and IL-8 than did those treated with CP. This finding demonstrates the beneficial effects of this intervention. The significant anti-inflammatory effects of 250 mg/kg *M. oleifera* leaf extract are attributed mainly to the presence of kaempferol-3-glucoside and chlorogenic acid and a prior study supported our findings^61^.

Histological and ultrastructural observations revealed that CP causes an increase in atretic follicles and a decrease in ovarian follicles. The cells exhibited apoptotic signs on the antral follicles and distorted granulosa cells with the appearance of a fibrotic corpus luteum and the findings of a previous study support our data^62^. *M. oleifera* leaf extract (150 and 250 mg/kg)—treated rats promoted follicle growth and maintained the oxidative balance. The mechanism by which *M. oleifera* leaf extract (250 mg/kg) protects ovarian and uterine tissues may involve reduced exposure to oxidative injury and decreased release of proinflammatory cytokines.

Among the limitations of our study is the lack of investigating the molecular mechanisms underlying the activity of *M. oleifera*, and evaluating the role of anti-Müllerian hormone and enzymatic antioxidants such as superoxide dismutase, catalase, and glutathione peroxidase.

## Conclusion

In the future, it is recommended to cover all the limitation of the study to gain a more valuable insight role of *M. oleifera* leaf extract test invivo studies and in randomized controlled clinical trials. The study shed the light on the protective effect of *M. oleifera* leaf extract against CP-induced reproductive dysfunction based on biochemical, hormonal, histopathological, and ultra- structural evidences. Our study demonstrated the potential use of 250 mg/kg *M. oleifera* leaf extract to prevent the noxious reproductive effects of CP through the presence of the most important antioxidant and phenolic compounds identified via HPLC analysis (gallic acid, p-hydroxybenzoic acid, chlorogenic acid, vanillic acid, rutin, rosmarinic acid and apegnin-7-glycoside.). These compounds reduce the ovarian concentration of MDA and increase the concentration of GSH in the ovary. In addition, *M. oleifera* leaf extract (250 mg/kg) had strong anti-inflammatory effects by reducing the concentrations of TNF-α and IL-8 in the ovaries. Consequently, there was a significant improvement in the hormonal results, histopathology and ultra- structure of the genital tract in rats treated with *M. oleifera* leaf extract (250 mg/kg). These findings are extremely important for paving the way for the use of *M. oleifera* leaf extract (250 mg/kg) as a nutritional and pharmaceutical supplement to prevent CP-induced ovarian damage and reproductive dysfunction.

## Data Availability

The datasets used and/or analysed during the current study available from the corresponding author on reasonable request.
